# The Biology and Control of the Greater Wax Moth, *Galleria mellonella*

**DOI:** 10.3390/insects8020061

**Published:** 2017-06-09

**Authors:** Charles A. Kwadha, George O. Ong’amo, Paul N. Ndegwa, Suresh K. Raina, Ayuka T. Fombong

**Affiliations:** 1 International Centre of Insect Physiology and Ecology (icipe), P.O. Box 30772-00100, Nairobi, Kenya; sraina@icipe.org (S.K.R.); fayuka@icipe.org (A.T.F.); 2 School of Biological Sciences, University of Nairobi, P.O. Box 30197-00100, Nairobi, Kenya; gongamo@uonbi.ac.ke (G.O.O.); pndegwa@uonbi.ac.ke (P.N.N.)

**Keywords:** honeybees, pests, *Galleria mellonella*, management, semiochemicals

## Abstract

The greater wax moth, *Galleria mellonella* Linnaeus, is a ubiquitous pest of the honeybee, *Apis mellifera* Linnaeus, and *Apis cerana* Fabricius. The greater wax moth larvae burrow into the edge of unsealed cells with pollen, bee brood, and honey through to the midrib of honeybee comb. Burrowing larvae leave behind masses of webs which causes galleriasis and later absconding of colonies. The damage caused by *G. mellonella* larvae is severe in tropical and sub-tropical regions, and is believed to be one of the contributing factors to the decline in both feral and wild honeybee populations. Previously, the pest was considered a nuisance in honeybee colonies, therefore, most studies have focused on the pest as a model for in vivo studies of toxicology and pathogenicity. It is currently widespread, especially in Africa, and the potential of transmitting honeybee viruses has raised legitimate concern, thus, there is need for more studies to find sustainable integrated management strategies. However, our knowledge of this pest is limited. This review provides an overview of the current knowledge on the biology, distribution, economic damage, and management options. In addition, we provide prospects that need consideration for better understanding and management of the pest.

## 1. Introduction

In the recent past, there has been accumulating empirical data that indicate declining trends in regional populations of both feral and wild honeybees [1,2,3,4,5]. This has spurred anxiety amongst apiculturists, scientists, and the general public due to the threat it poses to global food and nutritional security given the substantial contribution of honeybees to food production and the challenge of feeding the future global population with less resources [6,7,8]. This decline has been attributed to a myriad of interacting factors among which international trade in honeybee and hive products (which serves as a “carrier” of non-native species) [9], habitat loss and fragmentation [2,10], intensive application of pesticides [2,10,11], honeybee pests and pathogens [2,9,10,12], and genetic mismatches are considered key [2].

Honeybee pests are known to cause significant losses, and to transmit viral pathogens for which therapies remain nonexistent and continue to be challenging to eradicate. These disturbing trends have stimulated debates and remedial actions from the public, policy makers, and scientists which has resulted in unprecedented focus on pollinator health, especially those of honeybees. This has resulted in an upsurge in global honeybee research in a bid to provide both short- and long-term solutions that will ensure their survival and continual provision of pollination services [13]. A key step of this global effort is the production of a standardized manual for honeybee research called the COLOSS BEEBOOK [14]. The manual clearly highlights the disproportionate attention that various aspects of the bee health have received over the decades. One of such aspects is the ecology and management of the greater wax moth (GWM), *Galleria mellonella* Linnaeus, a severe pest of field-based honeybee colonies and stored combs [12,15,16]. This pest has received more attention as a model organism for toxicological investigations involving entomopathogenic organisms than as a honeybee pest, with more focus on proven (demonstrated) control measures [17,18,19]. However, with renewed interests in honeybee health and the moth’s increasingly recognized economic role globally, especially in Africa and Asia [9,20], there is an urgent need to advance our understanding of its biology and ecology with a special focus on both existing and potential management tools. This review brings together information on the biology, ecology, available management tools, and future research directions for management of GWM.

## 2. The Biology of the Greater Wax Moth, *G. mellonella*

### 2.1. Taxonomy

The term wax moth is a common name which refers to different species of moths that invade, attack, and damage honeybee colonies and hive products [16,19,21]. They are also known as the web (or wax) worm [21], the bee moth, or the wax (or bee) miller [19]. Included in the list are: the GWM (*G. mellonella*), the lesser wax moth (*Achroia grisella* Fabricius) [12,16,19,20], the Indian meal moth (*Plodia interpunctella* Hubner) [16], the bumble bee wax moth (*Aphomia sociella* Linnaeus) [16], and the Mediterranean flour moth (*Anagasta kuehniella* Zeller) [12].

*Galleria mellonella* is a member of the Galleriinae subfamily within the family Pyralidae of Lepidopteran order. Previously, the pest was classified as *Galleria cereana* by Fabricius and as *Galleria obliquella* by Walker [21]; it was later reclassified and named *G. mellonella* by Linnaeus [17,21]. A closely related species is the lesser wax moth, *A. grisella* [16,19,22]. As opposed to the GWM, the lesser wax moth is less destructive and less common [19]. However, both undergo complete metamorphosis. Key morphological features that are important in differentiating the two species can be found in a previous review article [19].

### 2.2. Morphology

Egg: Wax moth eggs vary in size, with an average length and width of 0.478 mm and 0.394 mm, respectively. The egg is of spheroidal shape with interspersed wavy lines which gives it a rough texture [19,21]. The egg’s colour varies from pink, through cream white to white, with little established knowledge on the mechanism(s) driving such colour changes.

Larva: Upon hatching, wax moth larvae are approximately 1–3 mm in length and 0.12–0.15 mm in diameter [21,23]. Prior to pupation, late instar larvae are about 25–30 mm in length and 5–7 mm in diameter. At the larval stage, sexing into male and female is not yet possible due to the absence of sex specific external morphological characters. The larva is polipod (eruciform), with six legs on the thorax and a number of prolegs on the third to sixth abdominal segments. The larva is cream white in colour, with its sclerotized body parts, although it darkens as it grows with each successful molt. The head is composed of three well developed apical teeth but lacks sub-apical teeth [23]. It might be possible that the apical teeth confer an adaptive advantage and thus contribute to the destructive nature of larvae. Though not conspicuous with the naked eye, retractable antennae are present and visible under light microscopy.

**Figure 1:**
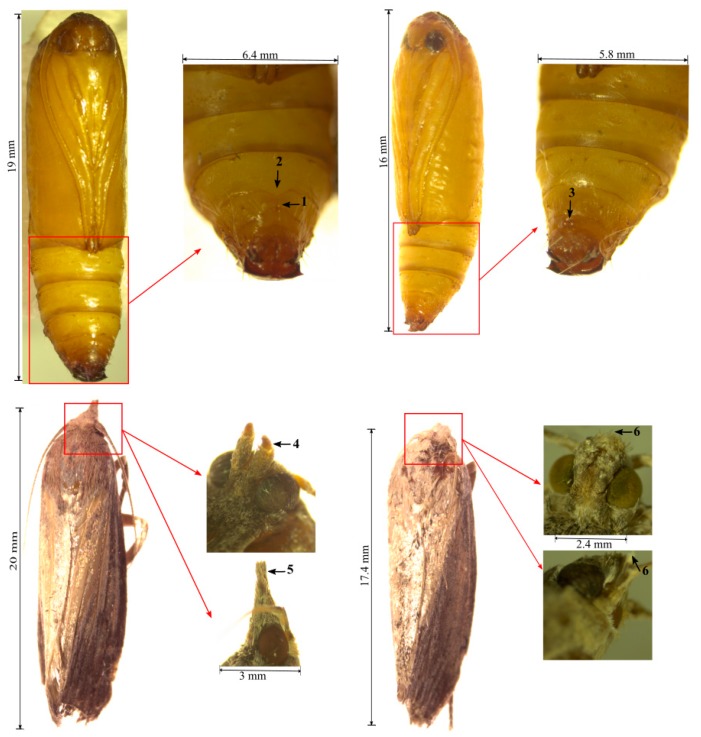
Key diagnostic features for the greater wax moth. (**a**) Female pupa; (1) and (2), cloven sterna forming copulatrix’s aperture, (**b**) male pupa, (3) a pair of small rounded knobs representing the phallomeres, (**c**) wax moth female adult, (4) bifurcated proboscis, (5) labial palps projecting forward (beak-like appearance), (**d**) wax moth male adult, (6) curved and inwardly hooked labial palps (snub-nose appearance). Please refer to Ellis, et al. [19] and Smith [23] for more description on egg and larval stages.

Pupa: The greater wax moth pupa averages 12–20 mm in length and 5–7 mm in diameter [19,21,23]. Female pupae are normally longer than the males. The pupa is of obtect type, with all its extremities glued to the body by a secretion produced during ecdysis. At the onset of pupation, the pupa is white to yellow in colour, but gradually changes to brown and later to dark brown with age and development. Sexual dimorphism is present in the pupa just as in the adult stage. The female pupa possesses a cloven sternum which represents an aperture for the bursa copulatrix on its eighth abdominal segment [23] (Figure 1a) while the male lacks this but instead possesses a small pair of external rounded knobs on the ventral side of the ninth (9th) abdominal segment which represent the phallomeres (Figure 1b). The pupa possesses a pair of prominent eyes and antennae are engrafted in mesowing. Pupation often takes place in spun cocoons covered with faecal pellets and frass and provided with an opening which serves as an exit for the eclosing adult [16].

Adult: The adult greater wax moth exhibits distinct sexual dimorphism. The female wax moth averages 15–20 mm in body length, 31 mm in wingspan, and 169 mg in weight [16,19,21]. The male is considerably smaller and less dark in colour compared to the females. The forewings for both sexes show varying intensities of pigmentation with the anterior two-thirds covered by scales that give it a uniformly darker pigmentation, compared to the posterior one-third which is a mixture of stripes of darker and lighter pigmentation [16,19]. Larval diets and the developmental duration have been observed to influence adult body coloration [24]. The female moth has an almost straight distal forewing margin as opposed to the scalloped (notched) wing margin in males’ border [16,23,24]. Additionally, the female possesses a forward projecting labial palp which give the mouthparts a beak-like appearance (“pointed nose”) (Figure 1c), whereas in males it is curved sharply upwards and hooked inwards and appears snub-nosed (Figure 1d) [16,23]. Despite their mouth parts, adult wax moths (both males and females) are not known to feed due to their rudimentary and bifurcated proboscis as opposed to the hollow tube-like proboscis in feeding lepidopteran adults [23,25]. Both males and females have the same antenna type, filiform, which differ in their number of segments (40–50 in males and 50–60 in females) [26]. The greater wax moth has a typical six-part lepidopteran leg consisting of the coxa, trochanter, femur, tibia, tarsus, and pre-tarsus [23]. Further, adult distinguishing features are found in the abdomen, where the ninth to eleventh segments have been modified into an aedeagus and ovipositor in male and female moths, respectively.

### 2.3. Life Cycle

The greater wax moth is a typical holometabolous insect and develops through four distinct life stages, namely, egg, larva, pupa, and adult. The duration taken by the moth to complete its life cycle varies from weeks to months and is affected by both biotic (intra-and interspecific) and abiotic factors [22,27,28]. Intraspecific factors which affect developmental duration and survival include competition for food [16,27] and cannibalism (of vulnerable early instars and pupae by late instars) [27]. Some examples of interspecific drivers include parasitoids, honeybees [21], and the small hive beetle. Diet quality has been demonstrated to affect larva development [29,30] and boost immunity, as nutrient deprived larvae became susceptible to *Candida albicans* Berhout [31]. Abiotic factors such as temperature and relative humidity are crucial to the entire life cycle. It has been shown that temperature averages of 29–33 °C are optimum for development [16,27,32]. Though no reports exist for suitable humidity ranges for wax moth development, our observations show that 29–33% relative humidity (RH) appears appropriate for survival [33]. These observations strongly support the moth’s ability to survive in the tropics and subtropics [12,16,24]. As a nocturnal insect, its peak time of activity falls between 18:00–24:00 hours, the first half of scotophase. Nielsen and Brister [34] observed that freshly eclosed male and female moths flew to nearby trees at the onset of scotophase possibly to mate, from where only females flew back to honeybee colonies. These females were gravid and were loaded with spermatophores full of eggs [34]. As opposed to most lepidopterans, GWM display unique mating behaviours. Male moths produce an acoustic sound from a tympanal organ to stimulate females which respond by fanning wings [35]. Males immediately releases a sex pheromone which ultimately attracts the females [26,35], resulting in mating. Males have a longer lifespan (ca. 21 days) than females (ca. 12 days) [32].

Oviposition begins a fairly short time after emergence and mating of females [21]. During oviposition, female moths lay eggs in clusters of 50–150 in tiny cracks or crevices inside the hive [12,16,19,24,28,36], which minimizes egg detection and enhances larval survival [28]. Eggs take between 3–30 days before hatching into larvae [16,24,28]. Gravid female moths are known to prefer strong colonies over weak counterparts as potential hosts [16]. Upon hatching, the wax moth larvae move from the cracks and crevices onto honeybee comb (natural diet) where they begin to feed and destroy the comb structure. The larvae feed on honey, pollen, and brood during which they display aggregation and cannibalism (only in case food shortage) [16,27]. Larvae can be maintained on artificial diet consisting of honey, wax, and cereal products [33]. More intense feeding is exhibited at early instars than at late instars [27]. Larvae undergo 8–10 moulting stages and spin silk threads across all stages but only the last instar spins a cocoon [28]. Larvae take between 28 days and 6 months before pupation. Paddock [21] and Nielsen and Brister [27] observed that larvae isolated from honeybee comb (food) appeared lost for a while but shortly proceeded in the direction of the food source, which is suggestive of the possible involvement of semiochemicals in larvae orientation towards food sources. The GWM pupa is immobile, does not feed, and is housed in cocoon. The pupal stage takes between 1–9 weeks [16,19,21]. GWM undergo between 4–6 generations annually. The greater wax moth enters reproductive diapause as an egg, larva, or pupa.

### 2.4. Distribution

**Figure 2:**
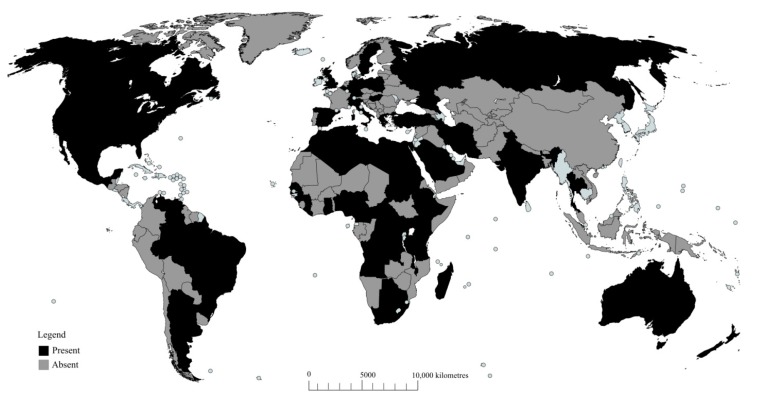
Global distribution of the greater wax moth (GWM) as of December 2016 in beekeeping regions in Africa [9,37,39,40,41,42,43,44,45,46], Asia [20,22,38,47,48,49,50,51,52], Australia [58], Europe [59,60,61,62], North America [12,16,21,27,34,53,54], Latin America [55,56,57], and island countries [63,64,65,66]. Areas in black (present) depict confirmed presence of GWM; areas in grey (absent) indicate no confirmed presence due to lack of literature.

The greater wax moth was first reported in honeybee colonies of Asian honeybee *Apis cerana* [21], but later spread to northern Africa, Great Britain, some parts of Europe, Northern America, and New Zealand [21,36]. However, Williams [16] and Shimanuki [12] later described the pests as ubiquitously distributed everywhere beekeeping is practiced. Presently, various reports [9,20,37,38] have emerged that might support Williams’ argument. Today, the presence of the greater wax moth has been confirmed in 27 African countries [9,37,39,40,41,42,43,44,45,46], nine Asian countries [20,22,38,47,48,49,50,51,52], five North American countries [12,16,21,27,34,53,54], three Latin American countries [55,56,57], Australia [58], ten European countries [59,60,61,62], and five island countries [63,64,65,66]. Even though there are some regions currently free of the pest, a recent case study in Kenya using futuristic scenario models has predicted the potential distribution of honeybee pests including the greater wax moth in ecological zones currently considered unsuitable for the pests [67]. Therefore, the pest’s distribution pattern is likely to change with the changing climatic factors. For an overview of the current global distribution of the pest and more references, see Figure 2.

## 3. Economic Importance of the Greater Wax Moth

The GWM is considered one of the most important pests of honeybee products owing to the destructive feeding habit of its larva [16,21,24]. The larva feeds on pollen, honey, wax, cast-off honeybee pupal skins, and brood, creates tunnels in the comb, and leaves masses of webs on the frame [12,16,27,68]. Damage occurs as the larvae create silk-lined tunnels through the hexagonal cell walls and over the comb surface. The tunnels and borings made by the larvae on the cell caps makes holes through which honey leaks out [24]. The silken threads entangles emergent bees, which as a result, die of starvation, a phenomenon described as galleriasis [16,22]. Moreover, large scale infestation of colonies by larvae of the greater wax moth often lead to colony loss, absconding, and reduction in the size of the migratory bee swarms [16,22]. Both the adults and larvae of GWM have been earmarked as potential vectors of pathogens [28]. For instance, faecal pellets of the larvae were found to contain spores of *Paenibacillus larvae* [28,69]. Recently, Israeli acute paralysis virus (IAPV) and black queen cell virus (BQCV) have been detected in larvae [51].

To date, an assessment of the economic impact of GWM at the global scale is still lacking. However, losses attributed to *G. mellonella* infestation in the southern United States were estimated to be approximately $3 and $4 million in 1973 and 1976, respectively, which approximately represent 3.9% and 5.1% of the profit in the respective years [69]. In the states of Florida and Texas, with tropical climatic conditions, approximately $5 and $1.5 losses per colony, respectively, were recorded in 1997 [69]. In Iran, the accumulated economic loss ascribed to *G. mellonella* infestation was estimated to be 38% (the author did not specify whether the percentage loss was based on the total input cost or other factors) [70]. The destructive nature of the pest is attributed to its high reproductive potential and rapid development time [12,33,68]. Considering the dynamic nature of the factors influencing growth and development of GWM and the fact that only Paddock [21] and Williams [16] have reported monetary losses due to GWM infestation, there is a need for the evaluation of economic losses both at regional and global levels.

## 4. Management of the Greater Wax Moth

Given the undesirable ecosystem outburst that ensued following excessive and continuous reliance on conventional pesticides [71], there have been considerable research efforts to provide alternative “reduced-risk products”. We focus here on the past and current management methods, and further highlight their merits and demerits which should form the basis of decision making for efficient control of GWM.

### 4.1. Cultural Practices

The most effective management of the GWM is by maintaining good sanitation [15,28]. This includes: keeping the colony strong and with adequate food sources [15,16,22,28], sealing cracks and crevices [15] (especially in regions inhabited by Asian honeybee, since they are poor propolisers) [22]. In addition, beekeepers should minimize pesticide application [15,16,22], replace combs regularly [28], and destroy infested combs showing signs of galleriasis [22]. Furthermore, there is a need for beekeepers to provide a proper storage system for hive products (such as wax, honey, and pollen stores) that are susceptible to attack by the pest and to protect colonies against pests and diseases [15,16,28]. These practices are easy to apply and pose no adverse effects to both honeybee colonies and non-target species. However, the cultural practices are tedious and only work best in small-scale beekeeping operations.

### 4.2. Temperature Control

Interruption of the developmental cycle of the GWM can be accomplished by exposing beekeeping equipment and bee combs to temperatures above (heating technique) or below (freezing technique) the tolerance range of the GWM [15,16,22,28]. In large-scale farming, infested combs are isolated from the hive, stacked together in insulated secondhand houses, and exposed to higher temperatures of approximately 45–80 °C for a period of 1–4 h [22], while for small-scale farming, the combs are kept in hot water for 3–5 h. However, it must be noted that heating sags and distorts wax. Infested combs can also be exposed to cold rooms or refrigerator equipment such as home freezers set at −7 °C to −15 °C for 2–4.5 h [22]. These techniques are advantageous, since growth and development of GWM is dependent on environmental factors such as temperature [22]. However, the techniques are only applicable in the absence of living honeybee stages and in small-scale beekeeping, since additional costs would be required for large-scale farming.

### 4.3. Chemical Control

Chemical fumigants are primarily used to manage the GWM in most beekeeping regions [15,16,28]. Fumigants that have been previously used and have been shown to be effective against wax moth include sulphur, acetic acid, ethylene bromide, calcium cyanide, methyl bromide, phosphine, paradichlorobenzene (PDB) naphthalene, and carbon dioxide [15,22,28]. Currently, only carbon dioxide is recommended for use as a fumigant. The fumigants were applied in stored combs under airtight conditions [22]. All fumigants targets and destroy all life stages of the moth, except PDB, which does not destroy eggs [22]. In addition, the application of fumigants is economically convenient and requires little knowledge of the biology of the wax moth [28]. However, the above listed fumigants (except carbon dioxide) pose health risks to the handler and lead to residues in hive products such as honey, rendering the product inconsumable [15]. More importantly, they are poisonous to honeybee colonies and non-target species, and are currently facing strong opposition in many countries where beekeeping is practiced [15,28].

### 4.4. Biological Control

Even though evidence for a successful and sustainable biological control agent of GWM is still lacking, previous researchers have explored various biological agents and bio-products including *Bacillus thuringiensis* Berliner (H-serotype V) (Bt), *Bracon hebetor* (Say), *Trichogramma* species, the red imported fire ant (RIFA) (*Solenopsis invicta* Buren and *Solenopsis germinita* Fabricius), and the use of the male sterile technique (MST).

#### 4.4.1. *Bacillus Thuringiensis* H Serotype

Controlling the GWM in live honeybee colonies has been challenging [15]. With the development of new techniques in the field of biotechnology, there was the potential of making a breakthrough using *Bacillus thuringiensis* (Bt) [15,28]. Infested combs are isolated and dipped into Bt. spore suspension or sprayed with the suspension while in the hive [17,22]. However, in a study conducted by Burges [72], Bt was only effective during the first season, but in the second and third seasons of trials, larval mortality dropped tremendously. In a separate study, Gulati and Kaushik [22] reported that Bt was only effective for 13 months. To explain this observation, Burges [72] argued that two reasons might have contributed to the seasonal decline in efficacy: (i) that during the maturity period, adult honeybees accumulate wax and propolis, and in effect, this diluted the spore content of Bt, and (ii) that the bacteria deteriorated. In addition, Williams [16] argued that the methods previously developed to apply spore formulations are not economically viable. Recently, in an effort to “supercharge” the efficacy of Bt, Basedow et al. [73] supplemented the biopesticide with neem; although their trials yielded higher larval mortality, no field evaluation on its practicability was reported. Therefore, it appears that these factors could have worked against Bt and hence contributed to its low adoption by commercial bee keepers [16,72,74]. Hanley et al. [75] recently reported significant mortality in GWM larvae fed with transgenic corn pollen (Cry1F). Moreover, incidences of lepidopteran resistance to Bt. toxins (Cry protein) have been reported in the family Pyralidae [76] and Plutellidae [77,78]. In addition, Dubovskiy et al. [79] reported a strain of GWM that had reduced the expression of genes involved in Cry protein binding, and as a result, renders Bt. ineffective against wax moths. The use of transgenic proteins is advantageous in that it (i) is species specific, (ii) reduces dependence on the application of conventional pesticides that are more broad spectrum and target both harmful and beneficial insect species, and (iii) can be easily applied either locally (per colony) or widely through feeders for supplemental feeding in large-scale beekeeping operations [28,80]. However, these advantages only apply if the transgenic pollen is not harmful to honeybees. A few cons to the use of transgenic proteins are: (i) that it may be unsafe for honeybee health, especially when the short -and long-term effects of both lethal and sub-lethal doses remain poorly studied [80,81,82,83,84,85], (ii) that its effects on other non-target hive arthropods such as the small hive beetle remain unknown, (iii) the lack of standardized protocols for assessing the effect of transgenic proteins on different life stages of the honeybee, and (iv) the ability of the target pest to develop resistance, as already observed by Dubovskiy et al. [79]. Thus, it is clear that the use of either entomopathogens or transgenics expressing their toxins remains a long shot.

#### 4.4.2. *Bracon hebetor* and *Apanteles galleriae*

*Bracon hebetor* Say (Hymenoptera: Braconidae) is a gregarious larval ectoparasitoid of many lepidopteran pests of the family Pyralidae [86]. Dweck et al. [87] showed that female *B. hebetor* utilize the male produced sex pheromone to locate the host, the GWM. Separately, in an attempt to determine the most suitable lepidopteran host for the parasitoid, Ghimire and Phillips [88] reported high oviposition rates but significantly lower parasitoid survival rates on *G. mellonella* larvae compared with other host species. However, there are no confirmed cases of field trials. Furthermore, *Apanteles galleriae* Wilkinson has been mentioned as a parasite of GWM larvae [21,22], but little is known about their interaction. Despite these findings, reports of where such parasitism takes place have been largely ignored. It is unlikely that such parasitism will take place inside a strong healthy colony, as the parasitoid is likely to be identified as an intruder and fended off. Also, for a day time insect, this parasitoid is likely to go through navigation challenges once inside the dark colony environment (assuming it gains access into a colony). With no reports of these having been reared out of naturally infested colonies (which did not collapse, as collapsed colonies are likely to be accessible by virtually any natural enemy), the potential of their use under field conditions is a little far-fetched. However, warehouses, holding poorly protected large wax quantities or wax products, could provide an avenue for their use. In addition, there are no confirmed cases of either closed indoor or open field trials of the parasitoid against the wax moth. Therefore, there is need to establish the ecological interaction between these parasitoids and the wax moth larvae, and between these parasitoids and honeybee colonies, so as to ascertain their usefulness. The latter must be approached cautiously, since other species of wasps have been reported as honeybee predators [22].

#### 4.4.3. *Trichogramma* Species

The GWM have been successfully used as a factitious lepidopteran host for mass rearing of the egg parasitoid *Trichogramma* species (*T. pretiosum* Riley, *T. evanescens* Westwood, and *T. minutum* Riley) [89]. However, the successful reports of egg parasitization are based on laboratory conditions, and use of laboratory stocks of different life stages of GWM, and thus may not accurately reflect its capacity to control natural population of GWM [69]. Therefore, with no evidence of natural parasitization of GWM eggs by *Trichogramma* species, similar ecological studies as earlier recommended are necessary to ascertain its potential as a control agent of GWM under indoor conditions.

#### 4.4.4. Red Imported Fire Ants (RIFA)

The red imported fire ants *Solenopsis invicta* and *Solenopsis germinita* feed on immature stages of GWM [16,21], but when evaluated as a biological control agent of the moth in stored super combs, *S. invicta* could only be effective in combination with promoted light and ventilation conditions [69]. Further, Paddock [21] argued that even though *S. germinita* was destructive under artificial conditions, no such observations have been recorded in or about apiaries. Therefore, it is possible that wax moth larvae are “immune” to fire ants under natural conditions. Despite its potential as a biological control agent, the cost of having large numbers of RIFA might raise genuine concerns because: (1) it is a predator of ground-nesting bees and (2) it is also a nursery pest which has infested an estimated 106 million ha of land in eastern states of the United States [69]. In North America, a significant decline in biodiversity of fauna has been linked with the invasion of natural habitats by RIFA [88]. These deficiencies strongly suggest that it will be inappropriate to use RIFA as a replacement for insecticides, and thus there is a need for alternative management options. In general, as Van Lenteren et al. [90] proposed, a successful release/application of a natural enemy requires an in-depth assessment and understanding of the environmental consequences of the natural enemy and ecological mechanisms required for its successful operation.

#### 4.4.5. Sterile Insect Technique (SIT)

Initially, SIT was used to eradicate screw-worm fly, *Cochliomyia hominivorax* Coquerel, through release of sterile males [91]. Following this control program’s success, it became a promising component of area-wide integrated pest management (IPM) for numerous lepidopteran pests [70,92,93]. However, up to the present date, only programs controlling for the pink bollworm moth, *Pectinophora gossypiella* (Saunders), and the codling moth, *C. pomonella* (Linnaeus), have benefited from this technique as part of their management programs [92]. Studies by Jafari et al. [70] revealed that male sterilization was most effective when wax moth pupae were partially sterilized (using 350 Gy of gamma-radiation). However, the release of irradiated pupae ended prematurely because the pupae were fragile and required a high input cost. In an effort to substitute irradiated pupae, irradiated eggs were released, but a similar experiment has never been performed on wax moths. In addition, Bloem et al. [92] showed that the emerging larvae were more destructive, raising fears that use of irradiated F1 eggs of GWM could exacerbate economic losses.

#### 4.4.6. Semiochemicals

Semiochemicals are chemical compounds that are released by living organisms into their environment, and which elicit either a behavioural or physiological response in a subsequent insect organism that perceives the signal. They are broadly classified into two groups: pheromones and allelochemicals [94,95]. In the past decade, there have been tremendous advancements in the chemical ecology of insects, following the invention of analytical and electrophysiological techniques, and equipment as well as improvement of existing ones [86,88,89,96,97,98,99,100,101]. Honeybees are among the most well studied living organisms [102]. They accumulate a variety of resources attractive to various arthropods including the small hive beetles, *Aethina tumida* Murray, the African large hive beetle, *Oplostomus haroldi* Witte and *O. fuligineus* Olivier, and the invasive mite, *Varroa destructor* Anderson & Trueman [102,103,104,105,106,107,108,109,110]. These invaders exploit hive resources as food for adults and immatures, and as oviposition sites [105]. More importantly, the invaders make use of the unique chemical cues associated with the hive to locate the host’s colonies [102,103,104,105,106,107,108,109,110]. However, little remains known on the chemical ecology of the GWM. Among the lepidoptera, the greater wax moth displays a unique pair-forming behaviour facilitated by sex-pheromones and acoustic signals produced by the males and perceived by the conspecific females. This is contrary to the general trend of female produced sex pheromones that attract conspecific males in lepidopterans [111,112].

Studies on mating systems in lepidoptera have focused more on the search for the adaptive significance of the stereotyped pair-formation and mating behaviours they display [113], with little attention on the unique mating behaviour in the GWM. In this section, we give an attempt to account for this. Spangler [35,114] pointed out that in *A. grisella* (another wax moth species), acoustic signals are utilized as a short-range cue that guides females to locate males. However, in GWM, the male produced acoustics initiates courtship, which then attracts virgin females to males. Once in close proximity, the male produces a sex pheromone that initiates mating. Over the past few decades, several authors have identified the sex pheromone components as nonanal, decanal, hexanal, heptanal, undecanal, and 6, 10, 14 trimethylpentacanol-2 and 5, 11-dimethylpentacosane [26,52,111,112,115]. Despite this knowledge, Flint and Merkle [112] reported that traps baited with the sex pheromone components (nonanal and decanal) were not attractive over long distances. These reports together suggest that in *G. mellonella*, acoustic signals and sex pheromone serve as long and short-range mating cues, respectively. The unique mating behaviour of the GWM could be of adaptive significance for the following possible reasons: (i) it allows the female to maximally invest most of her energy resources into reproductive processes such as egg production and location of a suitable oviposition site, as she does not produce the pheromones and acoustics needed to bring potential mates together, (ii) as the male sex pheromone serves as a kairomone for the generalist parasitoid (*B. hebetor*) [90], utilizing such a signal to locate the female and possibly her eggs/larvae may lead to lower success in foraging, and increase the survival chances of the wax moth’s progeny, (iii) the reliance on acoustics is advantageous because it can be transmitted on any substrate (except vacuum) compared to volatiles which can only be airborne, thereby increasing the range of transmission and perception while minimizing detection from possible natural enemies, (iv) the intensity and quality of the signals may inform the female of the quality of its potential mate and allow it to maximize its reproductive investments by only responding to perceived high quality males. However, these suggestions need to be substantiated through experimental investigations. Besides the short-range attraction of pheromone-baited traps, another constraint is their selective attraction for gravid virgins but not mated females. Thus, the incorporation of other signals attractive to the mated females could improve the effectiveness of such traps.

Also, Nielsen and Brister [34] observed that mated females preferred older honeybee combs over new ones. Additionally, several authors observed aggregation behaviour of the larvae [16,27]. Another observation made by Paddock [21] and Nielsen and Brister [27] was the orientation of larvae towards food sources, possibly indicating the role of food odours, an idea not discussed by these authors at the time. From these observations, it is obvious that the greater wax moth displays several behaviours that are likely to be mediated by semiochemicals. Larvae isolated from artificial diet and natural food were observed wandering for a while before crawling in the direction of the food sources. However, these authors did not speculate any reason behind such behaviours. In other lepidopteran species, for instance, the codling moth, *Cydia pomonella*, it was demonstrated that attraction to host and aggregation larvae is mediated by a host kairomone and an aggregation pheromone, respectively [116,117]. Therefore, it can be hypothesized that chemical cues induce these behaviours in the greater wax moth, thus calling for more chemoecological investigations into these behaviours. Currently we are investigating the role of semiochemicals in the behaviour of the GWM [33].

In summary, semiochemicals offers an opportunity to develop easily applicable, relatively cheap, and sustainable control management for *G. mellonella*. However, the effectiveness of traps baited with semiochemicals would be improved and better optimized by identification of minor components of the sex pheromone which might be playing a synergism role and incorporation of “acoustic like” sounds which apparently are very crucial for female attraction.

## 5. Future Prospects

After reviewing the present distribution and management practices of GWM, it is evident that the pest is both a field and store pest of honeybee colonies and stored bee combs (with and without pollen and honey, respectively), and that several research gaps exist. Specific areas of research with potential for the development of improved management and monitoring tools include:

- The potential of utilizing entomopathogens and baculoviruses as biological control agents. For instance, classical application of entomopathogenic nematodes (EPNs) has been applied to control other pests such as the Japanese beetle (*Popillia japonica* Newman) [118] and black vine weevil (*Otiorhynchus sulcatus* Fabricius) [119] and offers prospect of controlling the small hive beetle (*Aethina tumida*) [120,121]. Additionally, entomopathogenic fungi (EPFs) belonging to the genera Hyphomycetes, Zygomycota, and Deuteromycetes, have been significant against management of gypsy moth (*Lymantria dispar* Linnaeus) in the USA [122]. Recently, Dougherty et al. [123] reported the successful application of propagated baculovirus 1 against a GWM population. However, more studies are needed to optimize formulations and dosages of the virus. In addition, the reported cases of successful application of EPNs represents simple systems of pest-host interactions as opposed to the complex system of the GWM-honeybee colony interactions. Therefore, application of EPNs and EPFs against the GWM should probably focus on suppressing its population “outside” active bee hives, such as in storage facilities. In addition, future exploration of EPs should take into consideration the complex nature of honeybee colonies and the fact that some of these agents might present potential risks to life stages of colony members.
- Semiochemicals including pheromone, kairomone, repellents, and the development of semiochemical-based trapping systems. Such systems should take into consideration compositional variations in the chemical cues brought about by geographic disparities.
- Food and ovipositional baits. Volatiles released from food and oviposition sites of other pests dispensed either individually or in combination, have previously been shown to improve mass trapping [124,125,126]. Identification of such chemical signatures attractive to the greater wax moth would provide an opportunity to develop optimized trapping systems.
- Population modelling. Although the regional reports highlighted in this article are useful in understanding the current spread of GWM, little is known regarding the phenology of this pest in the future. Such knowledge gaps can be filled by using species distribution models (SDMs) [127] which take into account the interplay between the environmental and geographical variables. Recently, an ecological niche (EN) model was used to predict the future distribution of honeybee pests (including GWM) in Kenya [67]. We therefore recommend the application of such models to provide insight into the prospective geographical range of the pest, and in so doing, inform decision making in formulating precautionary measures to prevent and or minimize infestation of new colonies.

## 6. Conclusions

The greater wax moth continues to be a global challenge to the bee health and the beekeeping industry and the rather scanty research attention it has received compared to other global bee pests and parasites, such as *V. destructor* and *A. tumida*, clearly identifies it as good research niche. In conclusion, given the justifiable concerns over resistance issues to Bt. toxins, residual effects of chemical fumigants, and their deleterious effects to the non-target species and the environment, we advocate for more research towards developing enhanced trapping systems, biological management approaches, and cultural control practices. As these developments materialize, the stakeholders should strive to make control and management of GWM more integrated through IPM. Cultural practices (in Section 4.1) should form an integral component of the IPM program, especially in small-scale beekeeping operations as well as in situations where the estimated population of the pest is low. However, in similar large-scale operations and under high pest population densities, mass trapping techniques are recommended. As the focus is shifted to developing new techniques, spatial and temporal variations need to be considered, since they would likely affect the effectiveness of such techniques in different beekeeping regions. Notably, successful implementation of IPM requires coordinated efforts, especially by beekeepers in close proximity, because the possibility of a practice on an apiary having a negative impact on a nearby apiary cannot be ruled out in the case of repellent products. More importantly, there is no better indemnity against the scourge of the greater wax moth than to observe best apiary management practices that keep away the moth.
